# Downstream effects of polypathology on neurodegeneration of medial temporal lobe subregions

**DOI:** 10.1186/s40478-021-01225-3

**Published:** 2021-07-21

**Authors:** L. E. M. Wisse, S. Ravikumar, R. Ittyerah, S. Lim, J. Lane, M. L. Bedard, L. Xie, S. R. Das, T. Schuck, M. Grossman, E. B. Lee, M. D. Tisdall, K. Prabhakaran, J. A. Detre, G. Mizsei, J. Q. Trojanowski, E. Artacho-Pérula, M. M. de Iñiguez de Onzono Martin, M. M. Arroyo-Jiménez, M. Muñoz Lopez, F. J. Molina Romero, M. P. Marcos Rabal, S. Cebada Sánchez, J. C. Delgado González, C. de la Rosa Prieto, M. Córcoles Parada, D. A. Wolk, D. J. Irwin, R. Insausti, P. A. Yushkevich

**Affiliations:** 1 Department of Diagnostic Radiology, Lund University, Klinikgatan 13b, Lund, Sweden; 2 Department of Radiology, University of Pennsylvania, Philadelphia, USA; 3 Department of Pharmacology, University of North Carolina At Chapel Hill, Chapel Hill, USA; 4 Department of Neurology, University of Pennsylvania, Philadelphia, USA; 5 Center for Neurodegenerative Disease Research, University of Pennsylvania, Philadelphia, USA; 6 Human Neuroanatomy Laboratory, Neuromax CSIC Associated Unit, University of Castilla La Mancha, Albacete, Spain

## Abstract

The medial temporal lobe (MTL) is a nidus for neurodegenerative pathologies and therefore an important region in which to study polypathology. We investigated associations between neurodegenerative pathologies and the thickness of different MTL subregions measured using high-resolution post-mortem MRI. Tau, TAR DNA-binding protein 43 (TDP-43), amyloid-β and α-synuclein pathology were rated on a scale of 0 (absent)—3 (severe) in the hippocampus and entorhinal cortex (ERC) of 58 individuals with and without neurodegenerative diseases (median age 75.0 years, 60.3% male). Thickness measurements in ERC, Brodmann Area (BA) 35 and 36, parahippocampal cortex, subiculum, cornu ammonis (CA)1 and the stratum radiatum lacunosum moleculare (SRLM) were derived from 0.2 × 0.2 × 0.2 mm^3^ post-mortem MRI scans of excised MTL specimens from the contralateral hemisphere using a semi-automated approach. Spearman’s rank correlations were performed between neurodegenerative pathologies and thickness, correcting for age, sex and hemisphere, including all four proteinopathies in the model. We found significant associations of (1) TDP-43 with thickness in all subregions (r =  − 0.27 to r =  − 0.46), and (2) tau with BA35 (r =  − 0.31) and SRLM thickness (r =  − 0.33). In amyloid-β and TDP-43 negative cases, we found strong significant associations of tau with ERC (r =  − 0.40), BA35 (r =  − 0.55), subiculum (r =  − 0.42) and CA1 thickness (r =  − 0.47). This unique dataset shows widespread MTL atrophy in relation to TDP-43 pathology and atrophy in regions affected early in Braak stageing and tau pathology. Moreover, the strong association of tau with thickness in early Braak regions in the absence of amyloid-β suggests a role of Primary Age-Related Tauopathy in neurodegeneration.

**Supplementary Information:**

The online version contains supplementary material available at 10.1186/s40478-021-01225-3.

## Introduction

It has become increasingly evident that multiple neurodegenerative pathologies often co-occur in the brains of older adults and contribute to cognitive decline [31, 41, 55, 56]. In the presence of neurodegenerative disorders, such as pathologically confirmed Alzheimer’s disease (AD), other pathologies such as α-synuclein and TAR DNA-binding protein 43 (TDP-43) co-occur frequently [31, 55, 56]. These common neurodegenerative pathologies show a characteristic pattern of progression throughout the brain, where the medial temporal lobe (MTL) is frequently an early nidus [5, 7, 27, 46, 59], making the MTL an important region in which to study polypathology. For example, neurofibrillary tangle (NFT) pathology, a hallmark of AD, first accumulates in the transentorhinal region (which approximates Brodmann Area (BA) 35) and the lateral part of the entorhinal cortex (ERC), before spreading to the cornu ammonis 1 (CA1) subfield of the hippocampus, according to Braak and Braak [5].

In recent years, studies with both antemortem MRI and autopsy data have started to shed light on how different neurodegenerative pathologies are associated with different atrophy patterns in the brain and the MTL specifically [3, 15, 28, 29, 40, 46, 52, 66]. These studies enable the identification of patterns of neurodegeneration specific to each of the neurodegenerative pathologies. Since the field currently lacks reliable molecular in vivo biomarkers for TDP-43 or α-synuclein pathologies, incorporating proteinopathy-specific patterns of brain atrophy into structural MRI biomarkers offer a promising opportunity for detecting and monitoring these pathologies in vivo. Such patterns could also provide early AD imaging biomarkers for neurodegeneration that are more specific to Alzheimer’s Disease Neuropathological Change (ADNC) and less confounded by comorbid pathologies than current commonly used MRI biomarkers of neurodegeneration (e.g. whole hippocampal atrophy).

While previous studies combining antemortem MRI and neuropathology data are very valuable, they are hampered by relatively low image resolution precluding more granular measures of the MTL. Moreover, there is often a long time interval (often 1 year or greater) between the MRI and autopsy that can weaken the association between in vivo measures of neurodegeneration and the underlying neurodegenerative pathologies. In this study, we aimed to advance upon previous work by investigating the association between semi-quantitative measures of four primary neurodegenerative pathologies (i.e. amyloid-β plaques, tau, α-synuclein and TDP-43) and the thickness of different MTL subregions measured on ultra high resolution postmortem MRI in a large dataset of brain donors with a broad range of neurodegenerative diagnoses but also some without any neurodegenerative disease. As part of this analysis, we also study the association of tau pathology with MTL subregional thickness in the absence of amyloid-β plaques to interrogate Primary Age-related Tauopathy, or PART [10].

We hypothesize that tau pathology score will be associated with BA35, ERC and CA1 thickness both in the presence and absence of amyloid-β [5], but also in the stratum radiatum lacunosum moleculare (SRLM) of CA because this layer is an early target of neurofibrillary pathology [4]. We hypothesize that TDP-43 pathology score will be associated with ERC and subiculum thickness [47]. We do not expect strong associations with MTL thickness measures for amyloid-β plaques [18, 19, 21] and α-synuclein pathology [8, 9, 14, 15] based on previous literature. However, with our ultra high resolution images and subregional measurements, we might be able to pick up more subtle effects on MTL subregions.

## Methods

### Specimens and postmortem MRI

Brain hemispheres were obtained from 58 donors; 45 specimens from autopsies performed at the University of Pennsylvania Center for Neurodegenerative Disease Research (CNDR) and 13 specimens from the University of Castilla-La Mancha Human Neuroanatomy Laboratory (HNL). Human brain specimens were obtained in accordance with the University of Pennsylvania Institutional Review Board guidelines. Where possible, pre-consent during life and, in all cases, next-of-kin consent at death was given. CNDR hemispheres were fixed in 10% formalin solution for at least 30 days before extracting intact MTL blocks. HNL cases were fixed by perfusion with 4% paraformaldehyde through both carotid arteries. The blocks were then imaged on 9.4 tesla small-bore scanner (Varian, Palo Alto, CA) at 0.2 × 0.2 × 0.2 mm^3^ resolution [1]. Details of the imaging protocol are provided in the Supplementary Material.

For this study, we were unable to obtain MRI measures and clinical neuropathology scores in the same hemisphere for each specimen. Therefore, for all specimens imaging was done on the hemisphere contralateral to the neuropathological assessment. For CNDR, cases came from the Frontotemporal Dementia Center (FTDC) and the Alzheimer’s Disease Research Center (ADRC). For the cases from the FTDC, the less affected hemisphere was typically selected for imaging because of ongoing studies at CNDR that require the most affected hemisphere. For cases in which antemortem T2-weighted in vivo MRI was available, the hemisphere with the best in vivo scan quality was selected for imaging. For the remaining cases the hemisphere for imaging was selected at random.

### MTL thickness measurements

Measurements were performed in the ERC, BA35, BA36 (BA35 and BA36 together make up the perirhinal cortex), parahippocampal cortex (PHC), subiculum (SUB), CA1 and SRLM. We selected MTL regions that we could identify on postmortem MRI with sufficient certainty. While CA2 or CA3 would also have been interesting, these regions are difficult to identify on MRI and were therefore not included. We included the SRLM because this layer of CA can be identified on high resolution MRI and is an early target of neurofibrillary pathology [4] and has been shown to be thinner in clinical AD [1, 32]. Our SRLM label refers to the thin hypointense structure (also known as the “dark band” [16]) that can be identified reliably on T2-weighted MRI, which mainly reflects the strata lacunosum and moleculare and not the stratum radiatum (see Figure 6 in [16]).

**Fig. 1:**
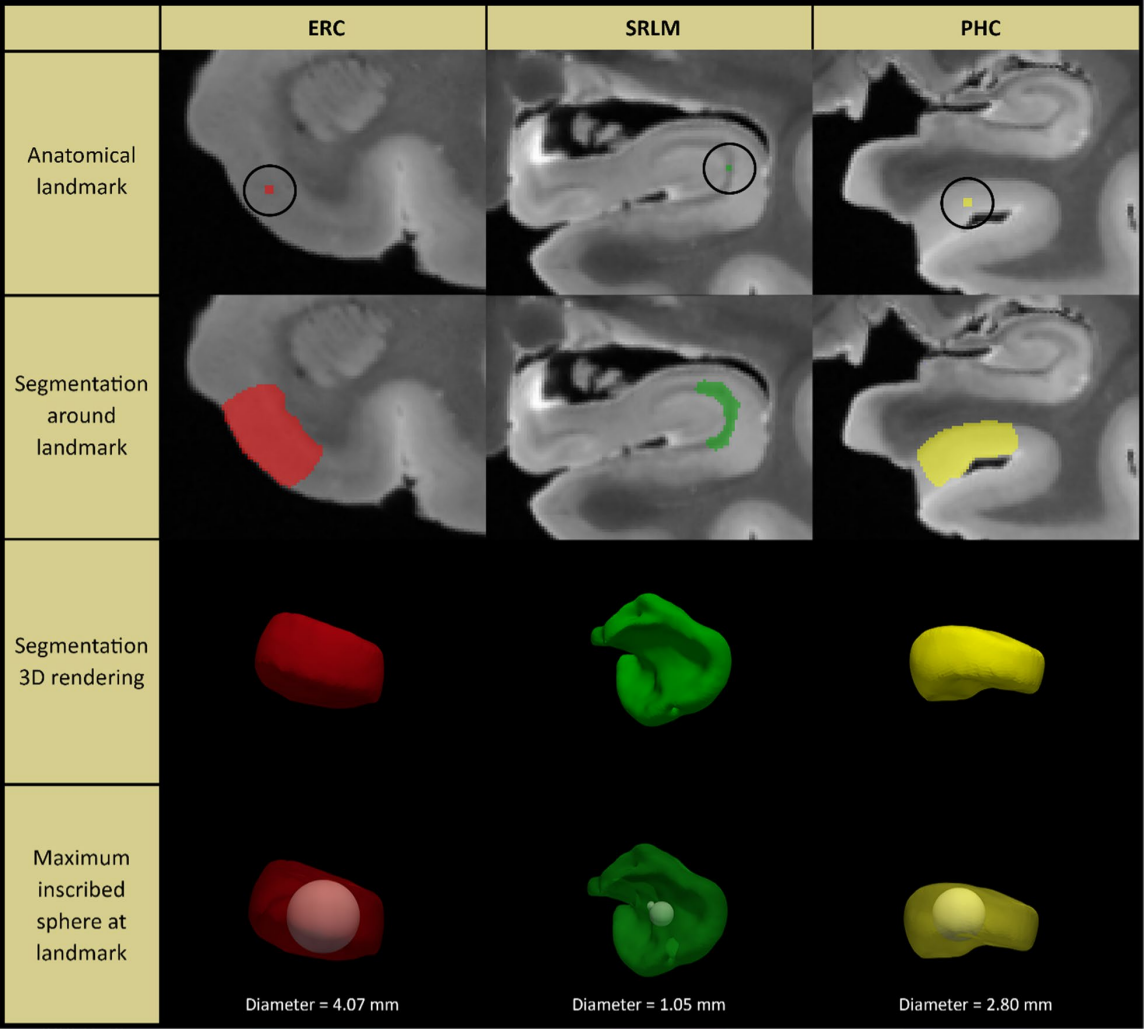
Method for obtaining thickness measures from high-resolution post-mortem MRI in medial temporal lobe subregions. For each anatomical location (indicated by a dot; first row), cortical thickness was measured by using semi-automated active contour segmentation in ITK-SNAP (with possible manual correction) to segment a portion of the surrounding gray matter extending ~ 5–10 mm from the dot in all directions (second and third row). Thickness at each location was measured as the diameter of the sphere overlapping the dot, fully contained in the gray matter segmentation, and having maximum possible radius (fourth row). Segmentations and fitted spheres were visually inspected for quality assurance. See supplementary Methods for details. Note that we only show three examples in this figure. For this study we performed thickness measurements for seven subregions and averaged thickness measurements over two locations for each subregion, see Additional file 1: Fig. 1. ERC = entorhinal cortex; SRLM = stratum radiatum lacunosum moleculare; PHC = parahippocampal cortex

For each subregion, two separate locations along the anterior–posterior axis of the MTL were identified to obtain thickness measurements, see Additional file 1: Fig. 1 and Supplementary Methods for more details. For each anatomical location (marked with a dot), cortical thickness was measured by using semi-automated active contour segmentation in ITK-SNAP [67] (with possible manual correction) to segment a portion of the surrounding gray matter extending ~ 5–10 mm from the dot in all directions (see Fig. 1). Thickness at each location was measured as the diameter of the sphere overlapping the dot, fully contained in the gray matter segmentation and with the maximum possible radius. Segmentations and fitted spheres were visually inspected for quality assurance. Thickness measures were then averaged over the two locations to obtain a single thickness measure per subregion. Thickness measurements could not always be performed for each location in each specimen, due to tears, other tissue damage or lack of contrast. We therefore reported the sample size for each subregion in the tables.

Note that we performed the thickness measurements in raw reconstructed MRI scans, in contrast to our previous paper [1], which is further explained in the Supplementary Material.

### Neuropathology measures

Analysis of the neuropathological data was performed at the University of Pennsylvania. Neuropathological assessments were performed by expert neuropathologists using established neuropathologic criteria for AD and related disorders [24, 39, 42, 45] using standard ordinal ratings scores. Neuropathology scoring has been established to be highly reliable between raters within and between laboratories [44]. Thirteen regions are routinely examined in the CNDR neuropathology evaluations as described in previous publications [60]. More precisely, tissue was embedded in paraffin blocks and cut into 6 μm sections for immunohistochemistry using the well-characterized primary antibodies and established methods (see [60] for more info): NAB228 (monoclonal antibody [mAb], 1:8000, generated in the CNDR) to detect amyloid-β deposits, phosphorylated tau PHF-1 (mAb, 1:1000, a gift from Dr. Peter Davies) to detect phosphorylated tau deposits, TAR5P-1D3 (mAb, 1:500, a gift from Dr. Manuela Neumann and Dr. E. Kremmer) to detect phosphorylated TDP-43 deposits and Syn303 (mAb, 1:16,000, generated in the CNDR) to detect the presence of pathological conformation of α-synuclein. The epitope specificity has been previously published in detail for the monoclonal antibodies used in this study. We use NAB228 that recognizes a N-terminal Aβ epitope that detects diffuse and neuritic plaque pathology [35], PHF-1 for tau phosphorylated at serine 396 and 404 [50], SYN303 for a disease specific conformation of alpha-synuclein [20] and ID3 for TDP-43 phosphorylated at residues 409/410 [48]. Each region was assigned a semiquantitative score i.e. none (0), rare (0.5), mild (1), moderate (2) or severe (3) for individual lesions (tau, amyloid-β, TDP-43 and α-synuclein pathologies). Note that the tau pathology score in this dataset represents multiple conformations of tau; it includes not only the 3R/4R tau typically associated with NFT, but also 3R and 4R tau associated with non-AD tauopathies. Assessment of pathology scoring has been well-established. For example, plaque scores are based on CERAD (Consortium to Establish a Registry for Alzheimer’s Disease) criteria [43]. Tau scores are based on the Braak staging scheme which describes not only the distribution but the severity of tauopathy across AD stages [6]. Scores for α-synuclein pathology are based on the McKeith criteria [42]. TDP-43 scoring was performed using criteria outlined by Mackenzie et al. [38]. All scores are established by examining sections at multiple levels of magnification to ensure adequate sampling of different regions of the section (at lower power) and ensuring accuracy (at higher power). An example of mild and severe pathology for each of the pathologies can be found in Additional file 1: Fig. 2. In an effort to preserve the scanned specimens for serial histopathology, these ratings were done by sampling the contralateral hemisphere. A composite score for the MTL was calculated by averaging the scores of CA1/SUB, ERC and dentate gyrus for each lesion of interest (tau, amyloid-β, TDP-43 and α-synuclein).

### Statistical analyses

Partial Spearman correlation analyses were performed with R package *ppcor*. Covariates included in the model were age, sex and hemisphere. Note that because of the relatively small size of this unique dataset, we did not correct for multiple comparisons.

## Results

### Demographics

Table 1 shows the demographics of the brain donor cohort and the subset who were considered amyloid-β negative (A-; defined by an A-score of 0 or 1). In brief, these 58 individuals had a median age of 75.0 years (range: 44–97) and 60.3% were male. Among the 35 A- donors, the average age was 73.0 years (range: 44–93) and 57.1% were male. Both the full cohort and the A- sub-cohort showed a wide range of neuropathological diagnoses. The MTL neuropathology scores as well as global neuropathological staging scores for AD neuropathological change (i.e. A, B and C scores [23]) are also reported in Table 1.

**Table 1:** Participant demographics

	Full dataset	A-subset
Number of specimens	58	35
Sex (% male)	60.3	57.1
Age (years) Median (range)	75.0 (44–97) years	73.0 (44–93) years
Mean ± SD	74.7 ± 11.3 years	72.0 ± 10.7 years
*MTL Tau score*		
Mean ± SD (range)	1.64 ± 0.93 (0–3)	1.34 ± 0.96 (0–3)
% score > 0 (N)	96.6% (56)	94.3% (33)
*MTL TDP-43 score*		
Mean ± SD (range)	0.47 ± 0.91 (0–3)	0.52 ± 0.99 (0–3)
% score > 0 (N)	27.6% (16)	25.7% (9)
*MTL amyloid-β scores*		
Mean ± SD (range)	0.92 ± 0.99 (0–3)	0.31 ± 0.59 (0–3)
% score > 0 (N)	63.8% (37)	40.0% (14)
*MTL α-synuclein score*		
Mean ± SD (range)	0.21 ± 0.54 (0–2.33)	0.08 ± 0.30 (0–1.67)
% score > 0 (N)	17.2% (10)	8.6% (3)
*Primary neuropathological diagnosis*		
None/limited pathology^a^	20.7% (8)	31.4% (7)
Intermediate-high ADNC	25.9% (15)	0% (0)
CBD	5.2% (3)	8.6% (3)
FTLD-TDP	13.8% (8)	20.0% (7)
LBD	8.6% (5)	2.9% (1)
Other^b^	13.8% (7)	17.1% (6)
PART	8.6% (5)	14.3% (5)
Pick’s disease	5.2% (3)	8.6% (3)
PSP	6.9% (4)	8.6% (3)
Two or more neuropathological diagnoses	70.7% (41)	65.7% (23)
*A score*		
0	25.9% (15)	42.9% (15)
1	34.5% (20)	57.1% (20)
2	8.6% (5)	0.0% (0)
3	31.0% (18)	0.0% (0)
*B score*		
0	22.4% (13)	37.1% (13)
1	29.0% (18)	42.9% (15)
2	20.0% (11)	14.3% (5)
3	21.0% (13)	0.0% (0)
Missing	11.3% (3)^b^	5.7% (2)^b^
*C score*		
0	55.% (32)	82.9 (29)
1	17.2% (10)	17.1% (6)
2	3.4% (2)	0.0% (0)
3	24.1% (14)	0.0% (0)

The MTL pathology scores shown in the table are the averages of the semi-quantitative ratings in the entorhinal cortex, cornu ammonis 1 and dentate gyrus from the hemisphere contralateral to the MRI scan

*MTL* medial temporal lobe, *TDP* TAR DNA-binding protein, *ADNC* Alzheimer’s disease neuropathological change, *CBD* corticobasal degeneration, *FTLD* frontotemporal lobar degeneration, *LBD* Lewy body disease, *PART* primary age-related tauopathy, *PSP* progressive supranuclear palsy. ^a^Also includes patients with low ADNC. ^b^B score was difficult to establish for some cases because they had primary tauopathies. ^b^”Other” includes the following primary neuropathological diagnoses: Amyotrophic Lateral Sclerosis (n = 1); Argyrophylic Grain Disease (n = 2); cerebrovascular disease (n = 1); multiple system atrophy (n = 1); other (n = 1); tauopathy unclassifiable (n = 1)

### Association of neurodegenerative pathologies with MTL subregional thickness measures

*Full dataset* Spearman partial correlations, corrected for age, sex and hemisphere, revealed a significant association between MTL tau pathology score and cortical thickness in regions affected early in Braak stageing, namely BA35, SRLM of the hippocampus, and at a trend level for ERC (Table 2). Note that the MTL tau pathology score in this dataset represents total burden of tau inclusions, including multiple conformations of tau. Widespread significant associations were also observed between MTL TDP-43 pathology score and all MTL subregional thickness measures. No significant associations with thickness were observed for amyloid-β or α-synuclein pathologies. Scatterplots of the pathology/thickness associations are shown in Additional file 1: Figs. 3–4.

**Table 2:** Partial Spearman correlations of semiquantitative MTL scores of neurodegenerative pathologies with MTL subregional thickness measures, including all pathologies in the same model

	ERC	BA35	BA36	PHC	SUB	CA1	SRLM
Sample size	55	53	53	56	58	58	57
Amyloid-β	0.10	0.11	− 0.02	− 0.18	0.05	0.05	0.16
Tau	− 0.26^a^	− **0**.31*	0.08	0.06	− 0.13	− 0.16	− **0**.33*
TDP-43	− **0**.44**	− **0**.46**	− **0**.35*	− **0**.45***	− 0.27^a^	− **0**.39**	− **0**.46***
α-synuclein	0.01	− 0.01	0.02	0.12	0.16	0.17	− 0.12

All models are corrected for age, sex and hemisphere

^a^*p* < 0.10; *p < 0.05; **p < 0.01; ***p < 0.001. Significant results are bolded. *TDP* TAR DNA-binding protein, *ERC* entorhinal cortex, *BA* Brodmann area, *PHC* parahippocampal cortex, *SUB* subiculum, *CA* cornu ammonis, *SRLM* stratum radiatum lacunosum moleculare

*Supplementary analyses in the full dataset* To investigate if tau and TDP-43 have a synergistic effect on neurodegeneration, we performed the analyses with an interaction term for MTL tau and TDP-43 scores. However, the interaction term was not significant for any region.

Since previous studies have shown that TDP-43 associations demonstrate an anterior-to-posterior gradient [22, 47], the analyses were repeated separately for anterior and posterior hippocampal subfield thickness measures, however no clear pattern arose (see Additional file 1: Table 1).

Third, to explore if there was a sex difference in the association of pathology ratings with thickness measures, we repeated all analyses that showed a significant association between pathology and thickness measure (see Table 2) including an interaction term with sex*pathology, but separately for tau and TDP-43 pathology. None of the interaction terms reached significance, except for tau*sex on BA35 (*p* = 0.006). Repeating the analyses separately for each sex showed that the association of tau pathology with BA35 thickness was stronger in females (correlation = -0.62; *p* = 0.003) than males (correlation =  − 0.11; *p* = 0.59), see Additional file 1: Fig. 5. Note that no significant differences were observed between males and females in BA35 thickness (females: 2.87 ± 0.63; males: 2.79 ± 0.43) or MTL tau ratings (females: 1.50 ± 0.88; males: 1.74 ± 0.96).

Fourth, to explore the effect of neuropathological diagnoses on the observed thickness-pathology analyses, Additional file 1: Figs. 6 and 7 illustrate the potential effect of diagnosis in different scatterplots. BA35 thickness is selected for these figures as an example, as the associations for tau and TDP-43 pathology were both strong for this specific subregion. The figures show different versions of the scatterplots for BA35 in Additional file 1: Figs. 3 and 4, each version highlighting cases that have one of the eight main neuropathological diagnoses, regardless of whether it was listed as a primary, secondary or tertiary neuropathological diagnosis. As can be seen in the figures, the different diagnoses do not seem to drive the observed association between BA35 thickness and either tau or TDP-43 pathology, except for frontotemporal lobar degeneration with TDP-43 inclusions (FTLD-TDP) which, as expected, partly seems to drive the association between TDP-43 and BA35 thickness.

*Amyloid*-*β negative subset* Table 3 shows that in A- subjects, MTL tau score was significantly associated with BA35 and at a trend level with ERC and CA1. In Additional file 1: Fig. 8 it can be observed that the association of MTL tau score with MTL structural measures may be obscured by individuals who have a low tau but high TDP-43 score. We therefore repeated the analyses in cases with low or absent levels of TDP-43 based on the MTL score in the hippocampus/ERC. The cut off was defined as < 0.5, based on inspection of the data where most subjects had a score of 0 or 0.17 and all other subjects had a score of 0.67 or higher. Significant associations were observed between MTL tau score and thickness of BA35, ERC, SUB and CA1 and at a trend level with SRLM. Scatterplots are shown in Additional file 1: Fig. 9.

**Table 3:** Partial Spearman correlations of semiquantitative MTL scores of neurodegenerative pathologies with MTL subregional thickness measures in different subgroups, including only tau in the model

	ERC	BA35	BA36	PHC	SUB	CA1	SRLM
Sample size	55	53	53	56	58	58	57
Tau in full dataset	− 0.27^a^	− **0**.30*	0.04	− 0.06	− 0.13	− 0.18	− **0**.31*
Sample size	33	31	32	34	35	35	34
Tau in A-subset	− 0.31^a^	− **0**.37*	− 0.06	0.14	− 0.22	− 0.30^a^	− 0.15
Sample size	25	23	24	26	27	27	26
Tau in A-/TDP-subset	− **0**.45*	− **0**.52*	− 0.08	− 0.09	− **0**.44*	− **0**.45*	− 0.38^a^

All models are corrected for age, sex and hemisphere

^a^*p* < 0.10; *p < 0.05; **p < 0.01; ***p < 0.001. Significant results are bolded. *ERC* entorhinal cortex, *BA* Brodmann area, *PHC* parahippocampal cortex, *SUB* subiculum, *CA* cornu ammonis, *SRLM* stratum radiatum lacunosum moleculare

## Discussion

This unique dataset combining measures of multiple neurodegenerative pathologies with ultra-high resolution post-mortem MRI in a large dataset of 58 brain specimens allowed us to investigate the association of frequently comorbid neurodegenerative pathologies with granular structural measures of the MTL, a hotspot for several neurodegenerative pathologies. We found widespread associations of MTL TDP-43 score with almost all MTL subregional thickness measures, whereas MTL tau score showed a more circumscribed pattern involving regions affected early in Braak stageing. In the absence of amyloid-β pathology, again strong associations of tau pathology were found with structural measures of regions affected early in Braak stageing.

Our results showing widespread MTL atrophy related to MTL TDP-43 score agree with two previous studies using antemortem MRI [3, 15]. This result is also in line with previous studies investigating specifically either the hippocampus or the amygdala [8, 30, 40, 58, 66]. There was one study by Wennberg et al. that did not report an association between TDP-43 and MTL structural measures [64], potentially because the participants in this study were clinically normal. No specific anterior-to-posterior gradient of atrophy in relation to TDP-43 was found in our study, which contrasts with the recent antemortem MRI study in an overlapping cohort of individuals [15]. This may be due to our cases being in more advanced stages of the disease or due to a difference in the selection of study subjects. The De Flores et al. study only included patients with intermediate to high ADNC [15], whereas our study included subjects with any neurodegenerative disease and likely had a higher percentage of subjects with FTLD-TDP. Notably TDP-43 pathology has been found to be associated with MTL atrophy both in the presence and absence of FTLD-TDP [3, 28, 40].

As previous studies have mostly analyzed gross MTL measures such as total hippocampal volume, the current study expands our knowledge by providing analyses of more granular MTL subregional measures. While TDP-43 pathology did not demonstrate associations with any specific MTL subregion, our dataset is growing and more quantitative measures for TDP-43 pathology are being generated [68] which in the future will allow for more fine-grained analyses or analyses in subgroups such as the recently introduced Limbic Age-related TDP-43 Encephalopathy, or LATE [46].

The atrophy pattern associated with tau pathology was more specifically restricted to regions affected early in Braak stageing: BA35, ERC (trend) and SRLM. Braak et al. identified BA35 (referred to as transentorhinal region in their report) and the lateral aspect of ERC as the earliest cortical site of tau NFT pathology [5]. Relatively early impact of NFT pathology in SRLM of CA, a region consisting of the apical dendrites [17], has also been reported [6]. Moreover, NFT pathology has long been associated with neuron loss in BA35 and ERC and neuropil loss in SRLM in multiple ex vivo studies [1, 18, 21, 61]. The current study provides the missing piece in the story of how tau pathology relates to structural MTL measures, complementing studies reporting associations between tau pathology and neuron loss [18, 21, 61], studies reporting associations between tau pathology and gross antemortem MRI measures such as total hippocampal volume [11] and more recently, studies reporting associations between in vivo biomarkers of tau pathology and MTL structural measures [12, 37, 62, 65]. This study confirms that the loss of neurons in granular regions in relation to tau pathology can be observed in measures that are more macrostructural than neuron loss, but still fine-grained, such as SRLM, ERC and BA35 thickness. This is a promising finding as these measures can also be obtained from high resolution in vivo MRI and could thus be used to detect early tau related pathology.

Moreover, we found preliminary evidence for a sex difference in the association between tau pathology and the earliest cortical region to be affected by NFT pathology, i.e. BA35, which may indicate that females may be less resilient to the effects of tau pathology. While this is a small sample to study sex differences and this finding is preliminary, it does fit with previous findings of higher atrophy rates in females [2] and a higher degree of metabolic dysfunction in the entorhinal cortex in response to tau pathology in females [53], compared to males. Although one previous study showed the opposite, that females showed more preservation of brain structure in the presence of tau [49]. We did not find higher levels of MTL tau pathology in females compared to males in contrast to previous studies [36, 51], which may be due to the small sample size in the current study. In general, this study further supports the importance of investigating sex differences in the pathogenesis of AD and future studies are required to further elucidate this.

We also found strong associations between MTL tau score and thickness in regions affected early in Braak stageing in the absence of amyloid-β, suggesting a role for PART in driving neurodegeneration. While associations between NFT pathology and volume loss of gross MTL regions measured on MRI have been reported before [29, 52], studies investigating this association have been very sparse. This is an important area of research, as the ramifications of PART are still unclear. In this study we provide further evidence that tau pathology in the absence of amyloid-β is not harmless and is in fact associated with structural changes in the MTL. Moreover, it extends previous work by providing a more fine-grained pattern of atrophy within the MTL, beyond total hippocampal volume. Our findings align well with recent in vivo studies showing associations between Tau-PET uptake and MTL structural measures in amyloid-β negative subjects ([12, 37] and provides support for the notion that PART may be underlying at least some Suspected non-Alzheimer’s pathophysiology (SNAP) cases [25, 26], a group of subjects who show AD-like neurodegeneration in the absence of amyloid-β. However, we note two studies where no association between Tau-PET uptake and MTL structural measures were reported in amyloid-β negative subjects [13, 63]).

It should be noted that the A- group in the current study actually includes a large variety of diagnoses (see Table 1) and the MTL tau score used in this study does not only reflect NFT pathology but also other conformations of tau (e.g. FTLD-Tau) [34]. However, the involvement of regions affected early in Braak stageing in our study are less implicated in FTLD-Tau [34], suggesting that age-related NFT pathology and not FTLD-Tau pathology is likely driving the observed associations. Additionally, repeating the analyses after excluding subjects with FTLD-Tau and Argyrophilic grain disease, we observed largely similar results, albeit weaker likely due to the smaller sample size (see Additional file 1: Table 2). Notably, the associations of MTL tau pathology score with MTL subregional thickness measures became stronger after excluding cases with high MTL TDP-43 scores. Indeed, as shown in Additional file 1: Fig. 5, some low tau/high TDP-43 cases may have obscured the associations between tau and structural measures. This finding has important implications for in vivo studies, for example, those investigating the associations between structural MRI and tau PET uptake or phosphorylated tau measured in cerebrospinal fluid, especially when these studies include the higher age range at which TDP-43 pathology is common [47].

The strengths of this study are the large sample size, the analysis of multiple pathologies and the granular assessment of 0.2 × 0.2 × 0.2 mm^3^ postmortem MRI. This study also has some limitations. The first limitation is the different fixation methods used at the CNDR and HNL (see section “Specimens and Postmortem MRI”). While fixation method may be a potential confounder in the thickness analyses, we could not correct for this in our analyses as fixation method is confounded by diagnosis, i.e. all non-neurological specimens are processed with a different fixation method than those with a dementia diagnosis. However, as all results regarding the associations of TDP-43 pathology and tau pathology are in line with the literature and as all brain specimens, regardless of fixation method, were fully fixed, we do not expect that this played an important role. Another limitation is that we did not correct for multiple comparisons because of the uniqueness of the data and the sample size. While this increases the chance of false positives, we believe that this issue was limited in this paper as our findings matched previous literature. A source of variability in postmortem studies assessing structural measures is brain swelling. We noticed one subject with severe brain swelling (when compared to the antemortem MRI, which was available in this subject). We re-ran the analyses without this subject but saw no notable differences in the results (see Additional file 1: Tables 3 and 4). A last limitation is that neuropathological scores were established in the contralateral hemisphere. While having neuropathological scores and thickness measurements in the same hemisphere would have been preferable, this data is not available in the current study as the imaged hemispheres are still undergoing additional processing, including dense sectioning which is a very labor intensive process. Moreover, this additional processing, as part of a larger study, includes different sectioning and staining methods in which we are less confident about the pathology score compared to what is typically done in the CNDR and other neuropathology centers. While inter-hemispheric differences have been observed for neurodegenerative pathologies, these do not seem to be systematic [33, 57] and we there do not expect this to have affected our results. Additionally, if anything, such inter-hemispheric differences in neuropathology severity may have decreased our power to detect an association.

In conclusion, this unique dataset revealed differential MTL subregional atrophy patterns with different neurodegenerative pathologies. While the current study is limited by the use of the semi-quantitative scores of the different pathologies in the contralateral hemisphere and thickness measurements at a small number of anatomical locations, we aim to improve on this in future work as we are collecting serial immunohistochemistry of these four pathologies in the same hemisphere as the MRI, and matching it to structural MRI, in a subset of the participants [54, 68]. This will allow for the creation of 3D neurodegenerative pathology maps which can be directly linked to local neurodegeneration. Moreover, as this dataset continues to grow, we will be able to further tease apart the effects of different neurodegenerative pathologies on MTL structure.

## Supplementary Information

**Additional file 1.** Supplementary Methods and Results.

## Data Availability

Anonymized data will be shared by request with any qualified investigator for purposes of validation and/or replication using our center’s established methods for sharing data.
