# Supplementary material for: Downstream effects of polypathology on neurodegeneration of medial temporal lobe subregions
Source: Acta Neuropathol Commun. 2021 Jul 21;9:128. doi: 10.1186/s40478-021-01225-3 (PMC8293481; doi:10.1186/s40478-021-01225-3)
Supplement: Supplementary file 1 — Additional file 1. Supplementary Methods and Results. [file 40478_2021_1225_MOESM1_ESM.docx]

**Supplementary Materials**

**Specimens and postmortem MRI**

Specimens were scanned in a cylindrical container with an MRI-neutral industrial lubricant Fomblin. The specimens were imaged with a custom-made 70 mm coil, using an acrylic holder with 49.8 mm inner diameter and a long (80 mm) z-field. Prior to 2017, specimens were imaged using a narrower 35 mm coil as described in Adler et al [1].

Prior to scanning, special precautions were taken to eliminate air bubbles trapped in both the brain and container. To reduce the chance of having air bubbles trapped along the cortical surface, the specimens were gently agitated by hand while submerged in the MRI-neutral lubricant. MRI scans were obtained using a multi-slice spin echo sequence. Sequence parameters varied slightly between specimens, with typical values being a repetition time (TR) of 9330ms, and an echo time (TE) of 23ms. Following image acquisition, the images were corrected for bias field non-uniformity using the N4ITK algorithm [3] and normalized to a common intensity range by clipping the intensities below the 0.1 and above the 99.9 percentile, and scaling the intensity range to [0,1000].

**Thickness measurements**

*Selecting thickness measurements:* For each subregion two locations were identified to perform the thickness measurement. LEMW was trained by RiI and placed dots to mark each location for each subregion. For the most anterior slice, a slice was selected at an anterior location of the amygdala, similar to the one shown in Supplementary Figure 1a. In this slice a dot was placed for entorhinal cortex (ERC), Brodmann Area (BA) 35 and 36. The dot for ERC was placed approximately in the middle of the width of the gyrus. For BA35 and BA36 the dot was placed dependent on the depth of the collateral sulcus, based on several examples which were created with the help of author RiI. Roughly, when the collateral sulcus had a depth of less than 5 mm, the dot for BA35 was placed 2/3 of the way along the medial bank of the collateral sulcus from the edge to the fundus, when between 6-10 mm, it was placed at the midpoint and when > 10 mm, 1/3 of the way along the medial bank. For BA36, when the collateral sulcus was less than 8 mm deep, the dot was placed at the midpoint of the lateral bank of the collateral sulcus, and when more than 8 mm, 2/3 of the way along the lateral bank of the collateral sulcus from the edge to the fundus.

The next slice for thickness measurements was an anterior slice of the hippocampus, generally anterior to the appearance of stratum radiatum lacunosum molecular (SRLM), see Supplementary Figure 1b. On this slice, dots for BA35 and BA36 were placed, again dependent on the depth of the collateral sulcus. Roughly, when the collateral sulcus was less than 5 mm, the dot for BA35 was placed 2/3 of the way along the medial bank of the collateral sulcus from the edge to the fundus, when between 5-10 mm, the dot was placed at the midpoint of the medial bank and when >10 mm, at 1/3 of the medial bank of the collateral sulcus from the edge to the fundus. For BA36, when the collateral sulcus was less than 5 mm, the dot was placed 1/3 of way along the lateral bank of the collateral sulcus from the edge to the fundus, when between 5-10 mm, the dot was placed at the midpoint of the lateral bank and when >10 mm, the dot was placed at 3/4 of the way along the lateral bank from the edge to the fundus.

The third slice represented a second anterior hippocampus slice where the digitations become fully visible and the most anterior dentate gyrus is visible, similar to the slice presented in Supplementary Figure 1c. In this slice, a dot for ERC was placed approximately in the middle of the width of the gyrus. The fourth slice was selected as being just anterior to the separation of the uncus from the rest of the hippocampus, see Supplementary Figure 1d. On this slice dots for subiculum (SUB), cornu ammonis (CA)1 and SRLM were placed. The dot for SUB was placed slightly medial of the width of the hippocampus. The dot for CA1 was placed in the lateral curve of the hippocampus and the dot for SRLM was placed adjacent to the dot of CA1. The fifth slice was selected as 6 mm after disappearance of the uncus, see Supplementary Figure 1e. The dots for SUB, CA1 and SRLM were placed on similar locations as described before. The dot for parahippocampal cortex (PHC) was placed approximately at the midpoint of the medial bank of the collateral sulcus. The sixth slice was selected as being just anterior to the appearance of the calcarine sulcus, see Supplementary Figure 1f. The second dot for PHC was placed in a similar location as described before.

*Thickness measurements:* For each anatomical location (marked with a dot), cortical thickness was measured in a semi-automated manner in ITK-SNAP. The “Active Contour Segmentation Mode” was entered after locating the dot of interest, and the MRI image was condensed to a bounding box includes the dot, surrounding tissue, and some background space. After the box was set, a small portion of the region of interest was then segmented manually. Once the region was manually segmented, another label was chosen and used to segment the surrounding tissue in order to distinguish this tissue from the region of interest. This was repeated for any adjacent background space. The classifier was then trained using these segmented areas. Once the training samples were found to be satisfactory, a bubble was then added to the location of the dot and the radius was altered to match the size of the tissue. Hereafter, the automatic segmentation was started and was allowed to run until the length of the small rectangular segmentation was about 1.5 times the width of the tissue. If the automated segmentation had any holes or extended into adjacent tissue that was not within the region of interest, manual edits were performed in all MRI slices of the segmentation.

As the performance of the thickness measurements was labor intensive, the thickness measurements were divided over two raters. At the start of the study, both raters performed segmentations in the same case and the thickness measurements of both raters showed a high correlation of 0.98 indicating high reliability.

We performed the thickness measurements in raw reconstructed MRI scans, in contrast to our previous paper, in which these scans were spatially transformed to correct for scanner gradient distortions [1]. Our approach to accounting for gradient distortion has changed over time. In Adler et al.[1], we performed a linear scaling (e.g., 6% in x, 3% in y, 11% in z) to correct for differences between the scanner coordinate frame and physical coordinate frame derived using a 3D printed phantom [4]. More recently, in addition to 9.4T scans, we began acquiring an accompanying 7T MRI scans, which are free of any scanner distortions and can therefore provide a more reliable ground truth for the physical coordinate space of each specimen. This is preferable since it is more accurate and allows for non-linear corrections. However, the distortion correction method is confounded by diagnosis, as all the specimens with non-neurological diagnoses use the latter correction method. We therefore decided to perform all analyses in uncorrected space instead of correcting for distortion correction in the statistical analyses. Even though the distortion will increase the noise in the measurements (as it is dependent on the location of the tissue in the scanner), this is unlikely to be related to pathology. To confirm that this did not affect our main findings, we performed 1) correlations between native and unwarped thickness measurements, 2) the analyses in the distortion corrected space, while correcting for the unwarping method, and 3) the same analyses in the subset of specimens that had a 7T scan, repeated with and without 7T-based distortion correction. The results of these analyses were very similar, suggesting that performing the analyses in uncorrected space did not notably affect our findings. See Supplementary Tables 5-7b.

**Supplementary Figure 1.** Example of the dot placements for each of the subregions. Screenshots of 0.2x0.2x0.2 mm^3^ post-mortem MRI images of the medial temporal lobe going from anterior (a) to posterior (f). The locations of the thickness measurements are indicated by the red dots in each of the figures. ERC=entorhinal cortex; BA=Brodmann Area; SUB=subiculum; CA=cornu ammonis; SRLM=stratum radiatum lacunosum moleculare; PHC=parahippocampal cortex


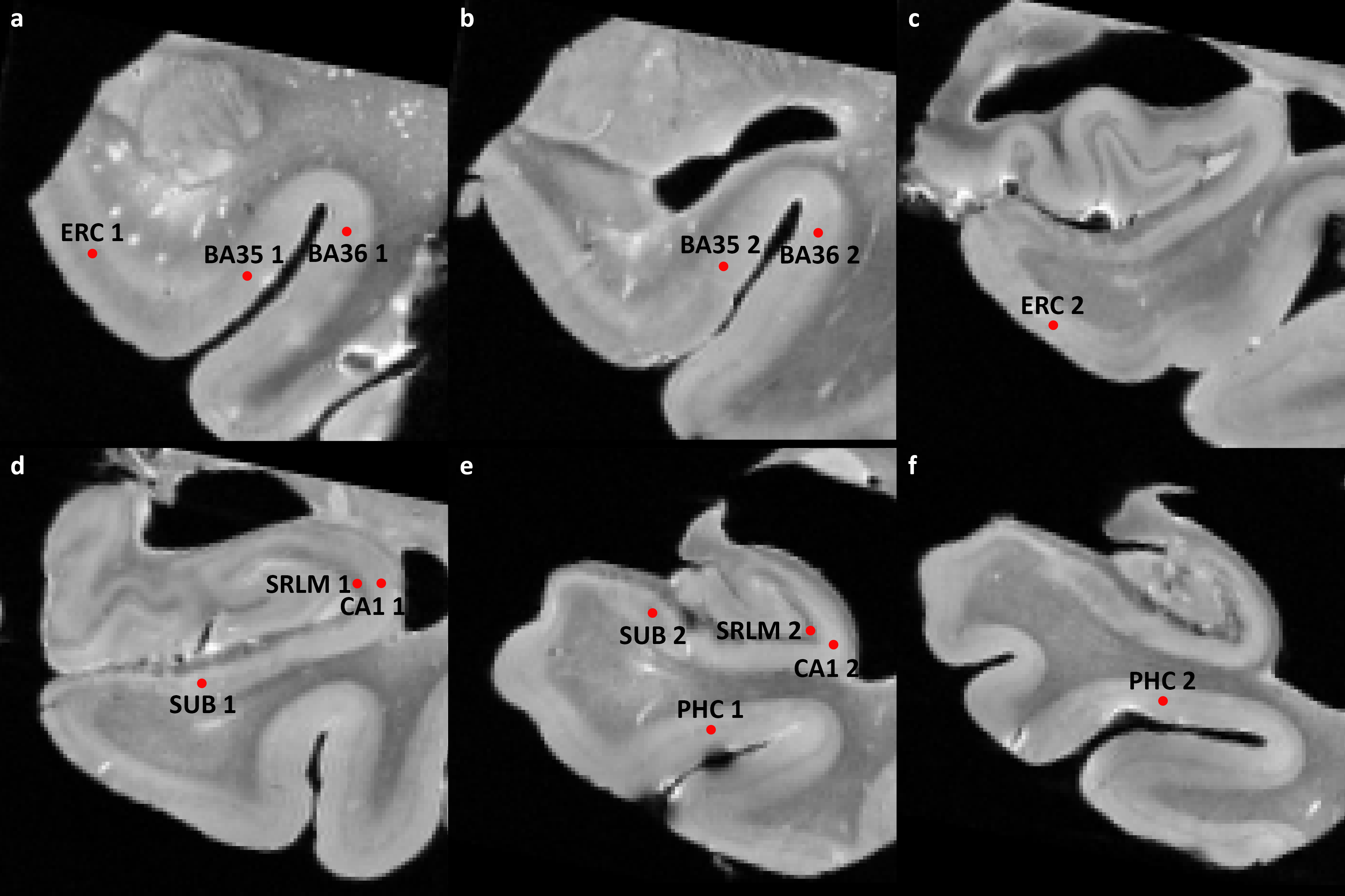


**Supplementary Figure 2.** Examples of pathological assessment for each pathological lesion in the medial temporal lobe. The upper panel shows mild ratings for inclusions and the lower panel shows severe ratings. The first column shows tau pathology from the entorhinal cortex (PHF-1 antibody), the second column shows amyloid-β plaques in CA1 (NAB228 antibody), the third column shows TDP-43 inclusions in the dentate gyrus (pS409/410 antibody), and the fourth column shows Lewy bodies (Syn303 antibody) in the entorhinal cortex. Scale bar is 100 *𝜇*m. This figure is reproduced with permission of John Wiley and Sons. The original version appeared in De Flores et al. [2].

**

**

**Supplementary Results**

**Supplementary Table 1.** Partial Spearman correlations of semiquantitative MTL scores of neurodegenerative pathologies with anterior vs posterior hippocampal subfield thickness measures. All pathologies are included in the same model. All models are corrected for age, sex and hemisphere.

|  | **Ant SUB** | **Post SUB** | **Ant CA1** | **Post CA1** | **Ant SRLM** | **Post SRLM** |
| --- | --- | --- | --- | --- | --- | --- |
| *Sample size* | *57* | *56* | *56* | *58* | *56* | *54* |
| Amyloid-β | 0.18 | 0.04 | 0.08 | 0.07 | **0.29*** | -0.01 |
| Tau | -0.08 | 0.04 | -0.19 | -0.05 | -0.24 | -0.23 |
| TDP-43 | -0.14 | -0.24^◊^ | **-0.42**** | -0.24^◊^ | **-0.27*** | **-0.45**** |
| α-synuclein | -0.01 | 0.08 | -0.03 | **0.27*** | -0.11 | -0.17 |

^◊^p<0.10; *p<0.05; **p<0.01; ***p<0.001. TDP=TAR DNA-binding Protein; Ant=Anterior; Post=posterior; SUB=subiculum; CA=cornu ammonis; SRLM=stratum radiatum lacunosum moleculare

**Supplementary Table 2.** Partial Spearman correlations of semiquantitative MTL scores of tau pathology with MTL subregional thickness measures in different subgroups. Subjects with a FTLD-Tau or Argyrophilic Grain Disease are excluded in these analyses. All models are corrected for age, sex and hemisphere.

|  | **ERC** | **BA35** | **BA36** | **PHC** | **SUB** | **CA1** | **SRLM** |
| --- | --- | --- | --- | --- | --- | --- | --- |
| *Sample size* | *46* | *44* | *44* | *45* | *46* | *46* | *45* |
| Tau in full dataset | -0.23 | **-0.37*** | 0.06 | -0.08 | -0.11 | -0.22 | -0.28^◊^ |
| *Sample size* | *25* | *23* | *24* | *25* | *35* | *25* | *24* |
| Tau in A- subset | -0.25^◊^ | -0.23 | 0.18 | 0.16 | -0.25 | -0.27 | -0.21 |
| *Sample size* | *18* | *16* | *17* | *18* | *18* | *18* | *17* |
| Tau in A-/TDP- subset | **-0.74**** | -0.43 | 0.20 | -0.05 | -0.45^◊^ | -0.35 | **-0.55*** |

^◊^p<0.10; *p<0.05; **p<0.01; ***p<0.001. ERC=entorhinal cortex; BA=Brodmann Area; PHC=parahippocampal cortex; SUB=subiculum; CA=cornu ammonis; SRLM=stratum radiatum lacunosum moleculare

**Supplementary Table 3.** Partial Spearman correlations of semiquantitative MTL scores of neurodegenerative pathologies with MTL subregional thickness measures, including all pathologies in the same model, excluding the subject with severe brain swelling. All models are corrected for age, sex and hemisphere.

|  | **ERC** | **BA35** | **BA36** | **PHC** | **SUB** | **CA1** | **SRLM** |
| --- | --- | --- | --- | --- | --- | --- | --- |
| *Sample size* | *54* | *52* | *52* | *55* | *57* | *57* | *56* |
| Amyloid-β | 0.11 | 0.12 | -0.07 | -0.20 | 0.08 | 0.06 | 0.14 |
| Tau | -0.26^◊^ | **-0.31*** | 0.14 | 0.09 | -0.16 | -0.18 | **-0.30*** |
| TDP-43 | **-0.44**** | **-0.46**** | **-0.39**** | **-0.46***** | -0.25^◊^ | **-0.38**** | **-0.47***** |
| α-synuclein | 0.01 | -0.01 | 0.001 | 0.12 | 0.17 | 0.18 | -0.13 |

^◊^p<0.10; *p<0.05; **p<0.01; ***p<0.001. TDP=TAR DNA-binding Protein; ERC=entorhinal cortex; BA=Brodmann Area; PHC=parahippocampal cortex; SUB=subiculum; CA=cornu ammonis; SRLM=stratum radiatum lacunosum moleculare

**Supplementary Table 4.** Partial Spearman correlations of semiquantitative MTL scores of neurodegenerative pathologies with MTL subregional thickness measures in different subgroups, including only tau in the model, excluding the subject with severe brain swelling. All models are corrected for age, sex and hemisphere.

|  | **ERC** | **BA35** | **BA36** | **PHC** | **SUB** | **CA1** | **SRLM** |
| --- | --- | --- | --- | --- | --- | --- | --- |
| *Sample size* | *54* | *52* | *52* | *55* | *57* | *57* | *56* |
| Tau in full dataset | -0.28^◊^ | **-0.31*** | 0.07 | -0.06 | -0.15 | -0.19 | **-0.29*** |
| *Sample size* | *32* | *30* | *31* | *33* | *34* | *34* | *33* |
| Tau in A- subset | -0.31^◊^ | **-0.40*** | 0.03 | 0.16 | -0.26 | -0.33^◊^ | -0.14 |
| *Sample size* | *24* | *22* | *23* | *25* | *26* | *26* | *25* |
| Tau in A-/TDP- subset | -0.43^◊^ | **-0.53*** | 0.08 | -0.08 | **-0.57**** | **-0.49*** | -0.40^◊^ |

^◊^p<0.10; *p<0.05; **p<0.01; ***p<0.001. ERC=entorhinal cortex; BA=Brodmann Area; PHC=parahippocampal cortex; SUB=subiculum; CA=cornu ammonis; SRLM=stratum radiatum lacunosum moleculare

**Supplementary Table 5.** Correlations between thickness measurements uncorrected for scanner gradient distortions and unwarped thickness measurements for each of the average thickness measurements. For specimens that also had 7T MRI (‘7T subset’), this modality was used to perform non-linear distortion correction. For the other (older) specimens (‘Affine subset’), linear correction based on a phantom was performed.

|  | **Affine subset** | | | **7T subset** | | |
| --- | --- | --- | --- | --- | --- | --- |
|  | **Sample size** | **Spearman correlation** | **p-value** | **Sample size** | **Spearman correlation** | **p-value** |
| *ERC* | 15 | 0.95 | <0.001 | 40 | 0.89 | <0.001 |
| *BA35* | 15 | 0.97 | <0.001 | 38 | 0.93 | <0.001 |
| *BA36* | 15 | 0.996 | <0.001 | 38 | 0.96 | <0.001 |
| *PHC* | 16 | 0.94 | <0.001 | 40 | 0.94 | <0.001 |
| *SUB* | 18 | 0.98 | <0.001 | 40 | 0.95 | <0.001 |
| *CA1* | 18 | 0.98 | <0.001 | 40 | 0.97 | <0.001 |
| *SRLM* | 18 | 0.97 | <0.001 | 39 | 0.94 | <0.001 |

ERC=entorhinal cortex; BA=Brodmann Area; PHC=parahippocampal cortex; SUB=subiculum; CA=cornu ammonis; SRLM=stratum radiatum lacunosum moleculare

**Supplementary Table 6a.** Partial Spearman correlations of semiquantitative MTL score of neurodegenerative pathologies with MTL subregional thickness measures derived by correcting MRI scans for gradient distortions. For specimens that also had 7T MRI, this modality was used to perform non-linear distortion correction (‘7T subset’), for the other (older) specimens, linear correction based on a phantom was performed (‘Affine subset’). All pathologies were included in the same model. All models were corrected for age, sex, hemisphere and unwarping method. Compare to Table 1 in the main paper, which uses thickness measures derived from uncorrected MRI scans.

|  | **ERC** | **BA35** | **BA36** | **PHC** | **SUB** | **CA1** | **SRLM** |
| --- | --- | --- | --- | --- | --- | --- | --- |
| *N Affine subset* | *15* | *15* | *15* | *16* | *18* | *18* | *18* |
| *N 7T subset* | *40* | *38* | *38* | *40* | *40* | *40* | *39* |
| Amyloid-β | 0.12 | -0.01 | -0.05 | -0.27^◊^ | 0.09 | 0.08 | 0.19^◊^ |
| Tau | -0.25^◊^ | -0.25^◊^ | 0.11 | 0.10 | -0.08 | -0.17 | **-0.30*** |
| TDP-43 | **-0.48***** | **-0.47**** | **-0.38**** | **-0.52***** | -0.18 | **-0.39**** | **-0.41**** |
| α-synuclein | 0.03 | -0.03 | 0.02 | 0.16 | 0.09 | 0.18 | -0.25 |

^◊^p<0.10; *p<0.05; **p<0.01; ***p<0.001. TDP=TAR DNA-binding Protein; ERC=entorhinal cortex; BA=Brodmann Area; PHC=parahippocampal cortex; SUB=subiculum; CA=cornu ammonis; SRLM=stratum radiatum lacunosum moleculare

**Supplementary Table 6b.** Comparison of the partial Spearman correlations between semiquantitative MTL scores of neurodegenerative pathologies and MTL subregional thickness measures derived using native (uncorrected) 9.4T MRI scans and scans corrected by warping to a 7T MRI scan, in a subset of specimens for which the 7T scans were available. All pathologies were included in the same model. All models are corrected for age, sex and hemisphere.

| **Native space data** | | | | | | | |
| --- | --- | --- | --- | --- | --- | --- | --- |
|  | **ERC** | **BA35** | **BA36** | **PHC** | **SUB** | **CA1** | **SRLM** |
| *Sample size* | *40* | *38* | *38* | *40* | *40* | *40* | *39* |
| Amyloid-β | -0.10 | 0.09 | 0.03 | **-0.39*** | -0.02 | 0.12 | 0.18 |
| Tau | -0.23 | -0.21 | 0.12 | -0.18 | -0.08 | -0.24 | -0.33^◊^ |
| TDP-43 | **-0.56***** | **-0.56***** | -0.29 | **-0.46**** | -0.28 | **-0.56***** | **-0.42*** |
| α-synuclein | -0.19 | -0.11 | 0.02 | 0.27 | 0.04 | 0.12 | -0.28 |
| **Unwarped data** | | | | | | | |
|  | **ERC** | **BA35** | **BA36** | **PHC** | **SUB** | **CA1** | **SRLM** |
| *Sample size* | *40* | *38* | *38* | *40* | *40* | *40* | *39* |
| Amyloid-β | -0.13 | -0.03 | -0.03 | **-0.40*** | 0.02 | 0.15 | 0.24 |
| Tau | -0.23 | -0.15 | 0.17 | 0.23 | -0.10 | -0.27 | **-0.38*** |
| TDP-43 | **-0.56***** | **-0.51**** | -0.30^◊^ | **-0.52**** | -0.23 | **-0.58***** | **-0.35*** |
| α-synuclein | -0.17 | -0.08 | -0.02 | 0.25 | 0.07 | 0.18 | **-0.36*** |

^◊^p<0.10; *p<0.05; **p<0.01; ***p<0.001. TDP=TAR DNA-binding Protein; ERC=entorhinal cortex; BA=Brodmann Area; PHC=parahippocampal cortex; SUB=subiculum; CA=cornu ammonis; SRLM=stratum radiatum lacunosum moleculare

**Supplementary Table 7a.** Partial Spearman correlations of semiquantitative MTL scores of neurodegenerative pathologies with MTL subregional thickness measures derived by correcting MRI scans for gradient distortions. For specimens that also had 7T MRI, this modality was used to perform non-linear distortion correction (‘7T subset’), for the other (older) specimens, linear correction based on a phantom was performed (‘Affine subset’). All models were corrected for age, sex, hemisphere and unwarping method. Compare to Table 2 in the main paper, which uses thickness measures derived from uncorrected MRI scans.

|  | **ERC** | **BA35** | **BA36** | **PHC** | **SUB** | **CA1** | **SRLM** |
| --- | --- | --- | --- | --- | --- | --- | --- |
| *N Affine subset* | *15* | 15 | *15* | *16* | *18* | *18* | *18* |
| *N 7T subset* | *40* | *38* | *38* | *40* | *40* | *40* | *39* |
| Tau in full dataset | -0.23 | -0.28^◊^ | 0.07 | -0.04 | -0.05 | -0.15 | -0.26^◊^ |
| *N Affine subset* | *8* | *8* | *8* | *9* | *10* | *10* | *10* |
| *N 7T subset* | *25* | *23* | *24* | *25* | *25* | *25* | *24* |
| Tau in A- subset | -0.33^◊^ | -0.32 | -0.12 | 0.19 | -0.07 | -0.25 | -0.04 |
| *N Affine subset* | *5* | *5* | *5* | *6* | *7* | *7* | *7* |
| *N 7T subset* | *20* | *18* | *19* | *20* | *20* | *20* | *19* |
| Tau in A-/TDP- subset | **-0.54*** | -0.39^◊^ | -0.12 | 0.03 | -0.29 | -0.33 | -0.36^◊^ |

^◊^p<0.10; *p<0.05; **p<0.01; ***p<0.001. ERC=entorhinal cortex; BA=Brodmann Area; PHC=parahippocampal cortex; SUB=subiculum; CA=cornu ammonis; SRLM=stratum radiatum lacunosum moleculare

**Supplementary Table 7b.** Comparison of the partial Spearman correlations between semiquantitative MTL scores of tau pathology and MTL subregional thickness measures derived using native (uncorrected) 9.4T MRI scans and scans corrected by warping to a 7T MRI scan, in a subset of specimens for which the 7T scans were available. All models were corrected for age, sex and hemisphere.

| **Native space data** | | | | | | | |
| --- | --- | --- | --- | --- | --- | --- | --- |
|  | **ERC** | **BA35** | **BA36** | **PHC** | **SUB** | **CA1** | **SRLM** |
| *Sample size* | *40* | *38* | *38* | *40* | *40* | *40* | *39* |
| Tau in full dataset | -0.26 | -0.22 | 0.11 | -0.05 | -0.13 | -0.26 | -0.31^◊^ |
| *Sample size* | *25* | *23* | *24* | *25* | *25* | *25* | *24* |
| Tau in A- subset | -0.26 | -0.26 | -0.18 | 0.01 | -0.18 | -0.37^◊^ | -0.10 |
| *Sample size* | *20* | *18* | *19* | *20* | *20* | *20* | *19* |
| Tau in A-/TDP- subset | -0.35 | **-0.56*** | -0.17 | 0.01 | -0.23 | -0.47^◊^ | -0.25 |
| **Unwarped data** | | | | | | | |
|  | **ERC** | **BA35** | **BA36** | **PHC** | **SUB** | **CA1** | **SRLM** |
| *Sample size* | *40* | *38* | *38* | *40* | *40* | *40* | *39* |
| Tau in full dataset | -0.25 | -0.21 | 0.13 | -0.02 | -0.13 | -0.26 | **-0.34*** |
| *Sample size* | *25* | *23* | *24* | *25* | *25* | *25* | *24* |
| Tau in A- subset | -0.33 | -0.21 | -0.15 | 0.07 | -0.22 | -0.34 | -0.17 |
| *Sample size* | *20* | *18* | *19* | *20* | *20* | *20* | *19* |
| Tau in A-/TDP- subset | **-0.51*** | -0.47^◊^ | -0.12 | 0.12 | -0.31 | -0.39 | -0.35 |

^◊^p<0.10; *p<0.05; **p<0.01; ***p<0.001. ERC=entorhinal cortex; BA=Brodmann Area; PHC=parahippocampal cortex; SUB=subiculum; CA=cornu ammonis; SRLM=stratum radiatum lacunosum moleculare

**Supplementary Figure 3.** Scatterplots of the association of MTL TDP-43 pathology with MTL subregional thickness measures in the full dataset. Note that the neuropathology scores can also fall in-between 0-0.5, 0.5-1, 1-2 and 2-3 because the scores are averaged over different MTL subregions. TDP=TAR DNA-binding Protein; ERC=entorhinal cortex; BA=Brodmann Area; PHC=parahippocampal cortex; SUB=subiculum; CA=cornu ammonis; SRLM=stratum radiatum lacunosum moleculare.

**
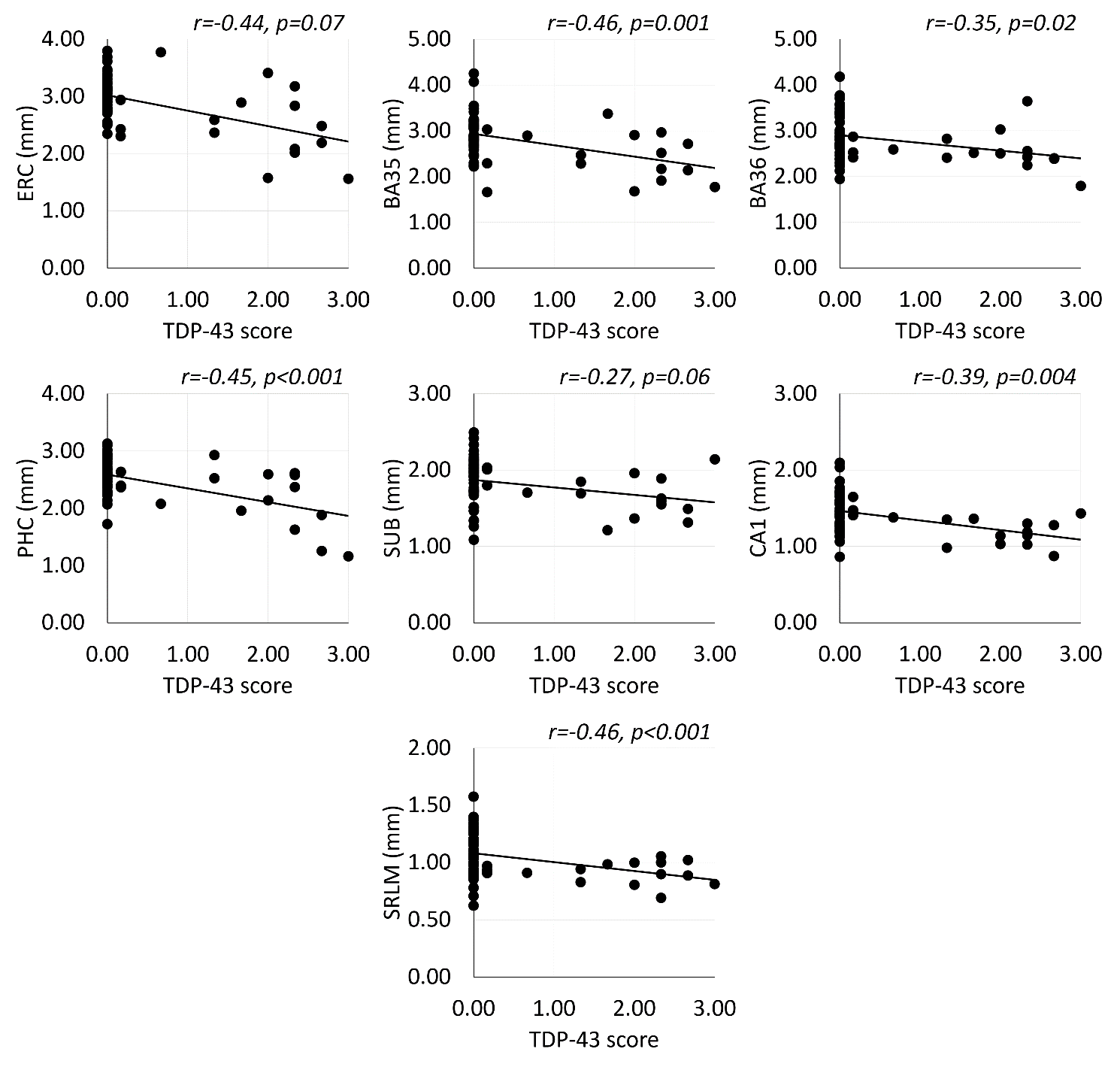
**

**Supplementary Figure 4.** Scatterplots of the association of MTL tau pathology with MTL subregional thickness measures in the full dataset. Note that the neuropathology scores can also fall in-between 0-0.5, 0.5-1, 1-2 and 2-3 because the scores are averaged over different MTL subregions. ERC=entorhinal cortex; BA=Brodmann Area; PHC=parahippocampal cortex; SUB=subiculum; CA=cornu ammonis; SRLM=stratum radiatum lacunosum moleculare.


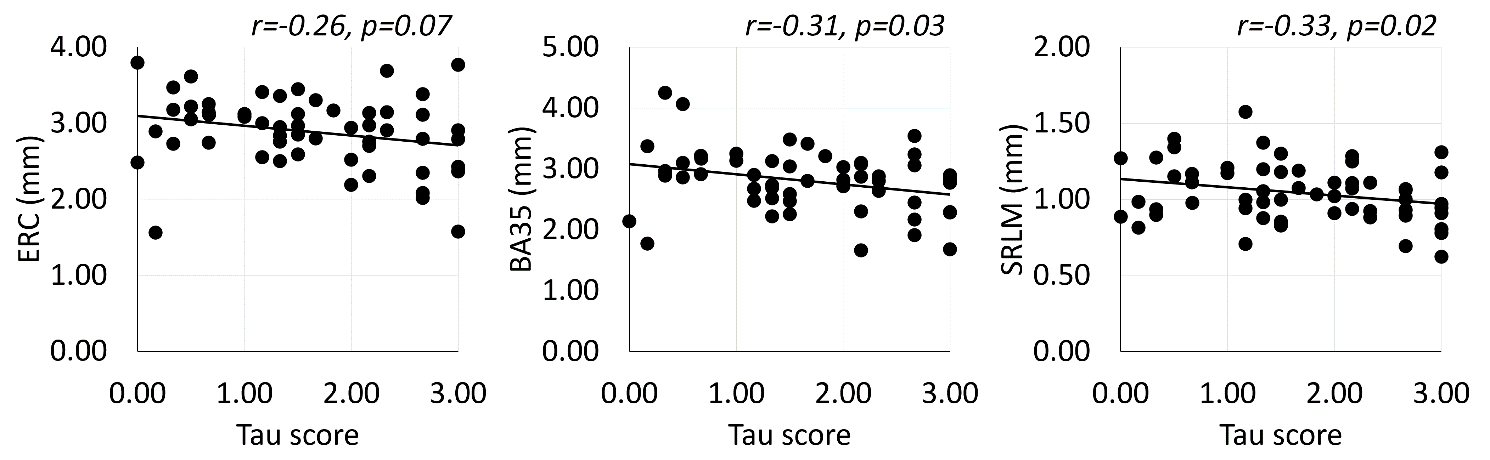


**Supplementary Figure 5.** Scatterplots of the association of MTL tau pathology with BA35 thickness measures in the full dataset separately for women and men. Note that the neuropathology scores can also fall in-between 0-0.5, 0.5-1, 1-2 and 2-3 because the scores are averaged over different MTL subregions. BA=Brodmann Area.


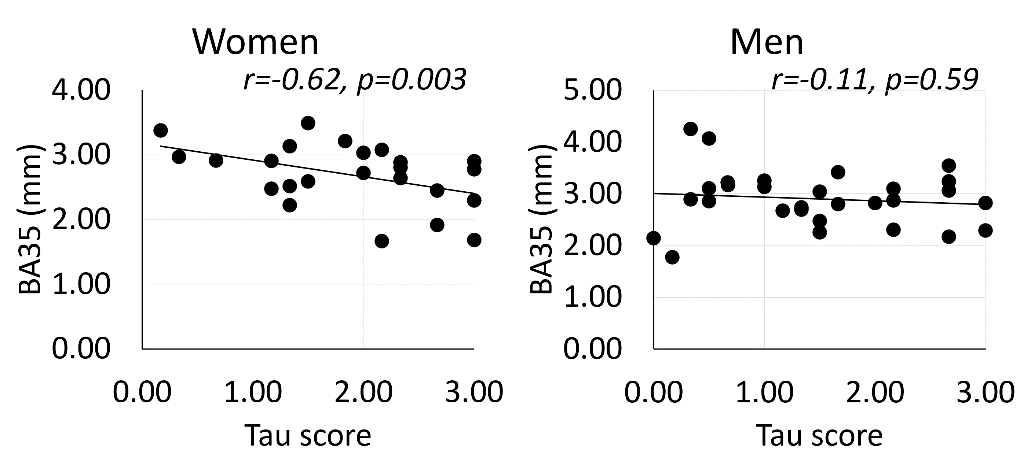


**Supplementary Figure 6.** Scatterplots of the association of tau pathology score with BA35 thickness contrasted for different neuropathological diagnoses (including primary, secondary and tertiary diagnoses). As can be seen, the different diagnoses do not seem to be driving the reported association between BA35 thickness and tau pathology score. Note that the total number of subjects with a certain diagnosis does not necessarily match those reported in Table 1 in the manuscript as secondary and tertiary diagnoses were also considered here and because for some subjects the BA35 thickness measurement was missing.

BA=Brodmann Area; ADNC=Alzheimer’s disease neuropathological change; CBD=corticobasal degeneration; CVD=cerebrovascular disease; FTLD-TDP=frontotemporal lobar degeneration with TAR DNA-binding Protein inclusions; int=intermediate; LBD=Lewy body disease; PART=Primary Age-Related Tauopathy; PSP=progressive supranuclear palsy.


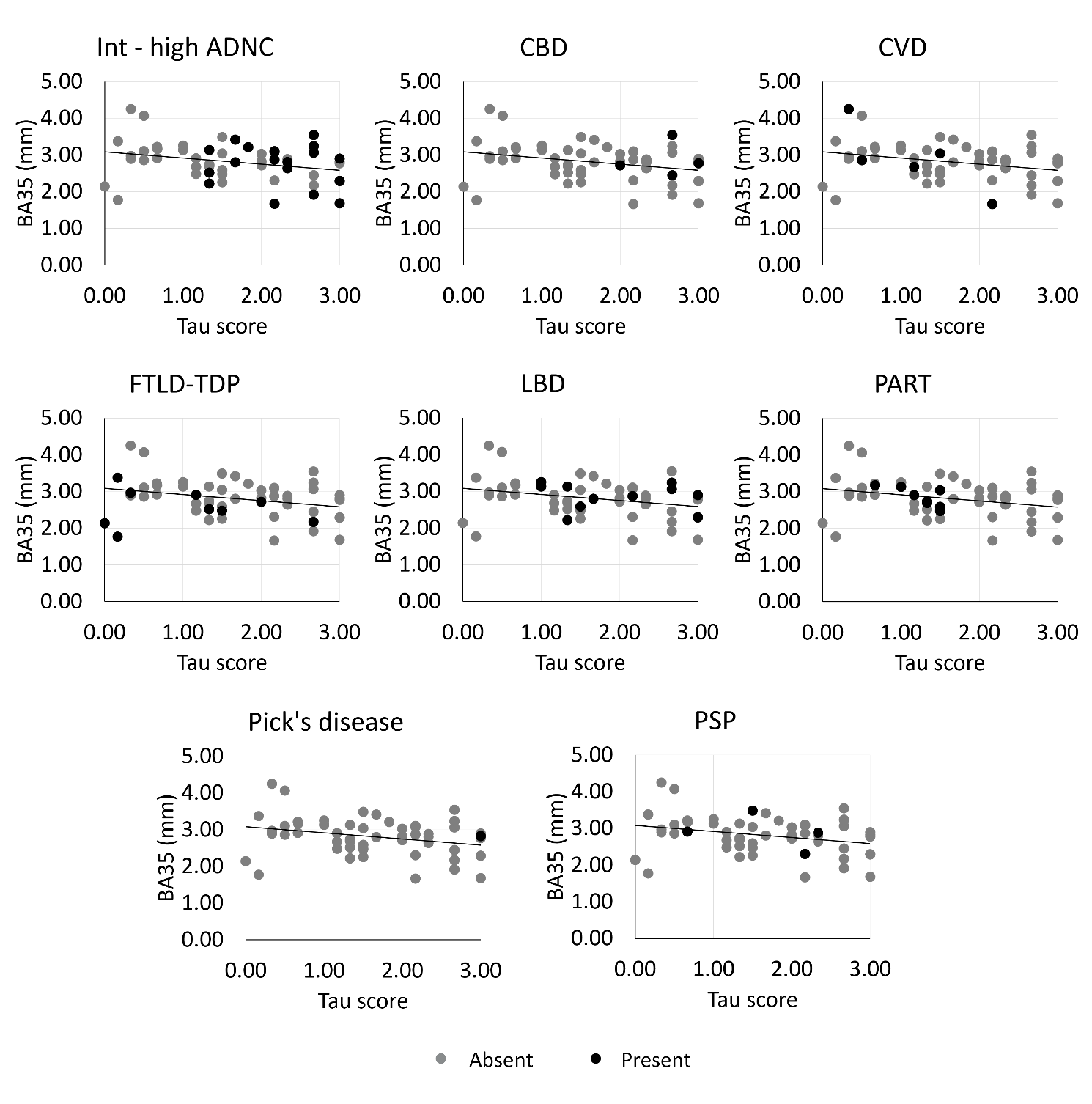


**Supplementary Figure 7.** Scatterplots of the association of TDP-43 pathology score with BA35 thickness contrasted for different neuropathological diagnoses (including primary, secondary and tertiary diagnoses). As can be seen, the different diagnoses do not seem to be driving the reported association between BA35 thickness and TDP-43 pathology score, except for FTLD-TDP which is to be expected. Note that the total number of subjects with a certain diagnosis does not necessarily match those reported in Table 1 in the manuscript as secondary and tertiary diagnoses were also considered here and because for some subjects the BA35 thickness measurement was missing.

BA=Brodmann Area; ADNC=Alzheimer’s disease neuropathological change; CBD=corticobasal degeneration; CVD=cerebrovascular disease; FTLD-TDP=frontotemporal lobar degeneration with TAR DNA-binding Protein inclusions; int=intermediate; LBD=Lewy body disease; PART=Primary Age-Related Tauopathy; PSP=progressive supranuclear palsy; TDP=TAR DNA-binding Protein.


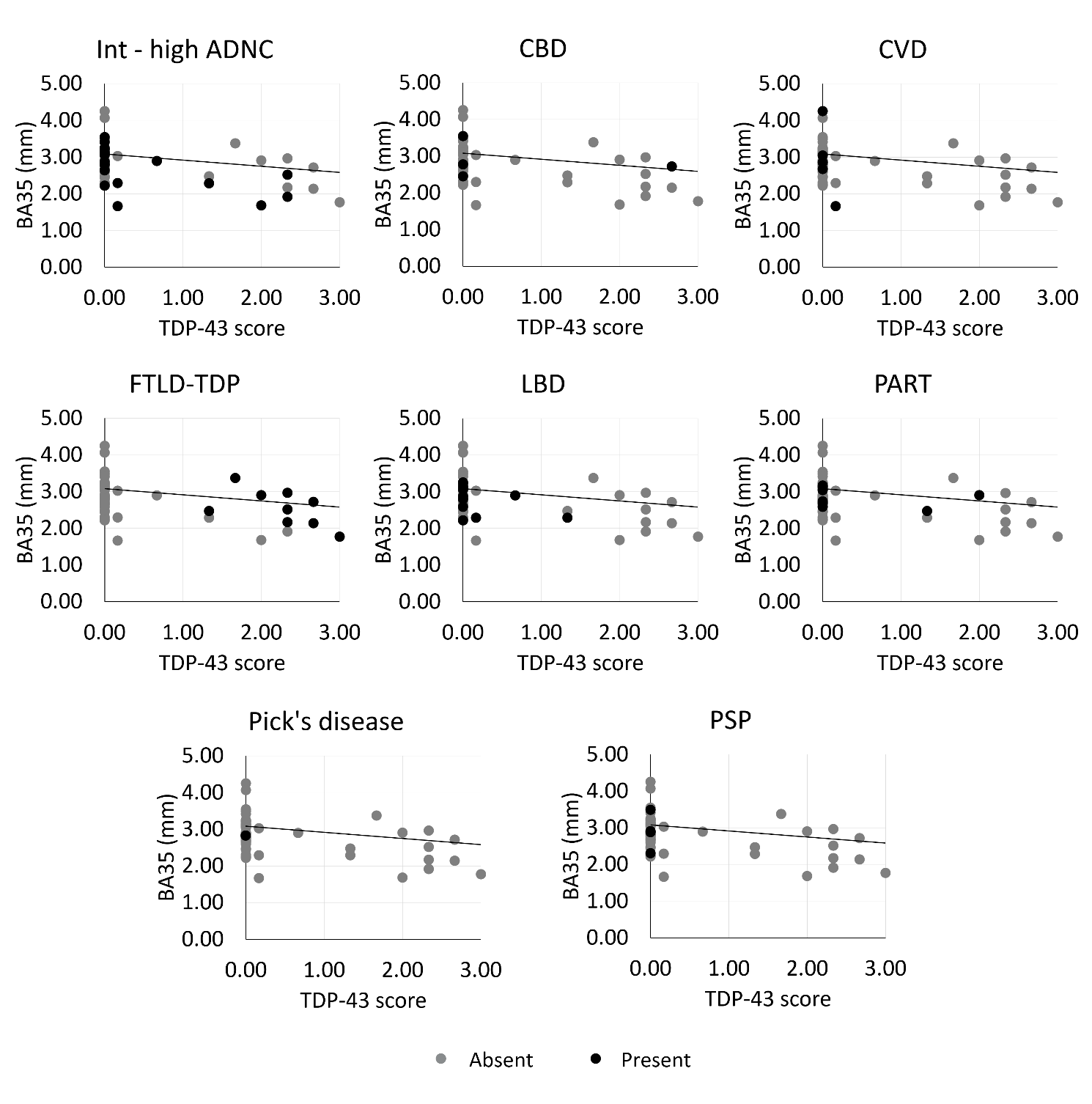


**Supplementary Figure 8.** Scatterplots of the association of MTL tau pathology with MTL subregional thickness measures in the amyloid-β negative subset. As can be observed in the scatterplots for the ERC and BA35, some cases with low MTL tau but high TDP-43 pathology (left lower corner of the graph) may be diluting the association between MTL tau and MTL thickness measures. Note that the neuropathology scores can also fall in-between 0-0.5, 0.5-1, 1-2 and 2-3 because the scores are averaged over different MTL subregions. TDP=TAR DNA-binding Protein; ERC=entorhinal cortex; BA=Brodmann Area; PHC=parahippocampal cortex; SUB=subiculum; CA=cornu ammonis; SRLM=stratum radiatum lacunosum moleculare.

**
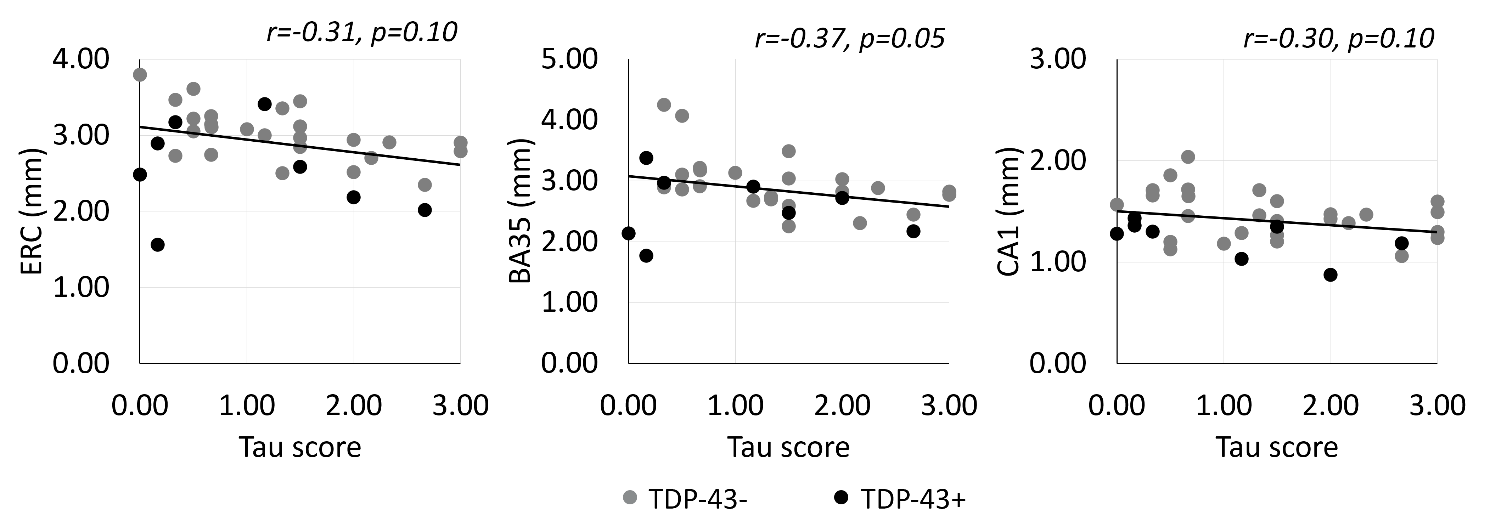
**

**Supplementary Figure 9.** Scatterplots of the association of MTL tau pathology with MTL subregional thickness measures in the amyloid-β negative and TDP-43 negative subset. Note that the neuropathology scores can also fall in-between 0-0.5, 0.5-1, 1-2 and 2-3 because the scores are averaged over different MTL subregions. TDP=TAR DNA-binding Protein; ERC=entorhinal cortex; BA=Brodmann Area; PHC=parahippocampal cortex; SUB=subiculum; CA=cornu ammonis; SRLM=stratum radiatum lacunosum moleculare.


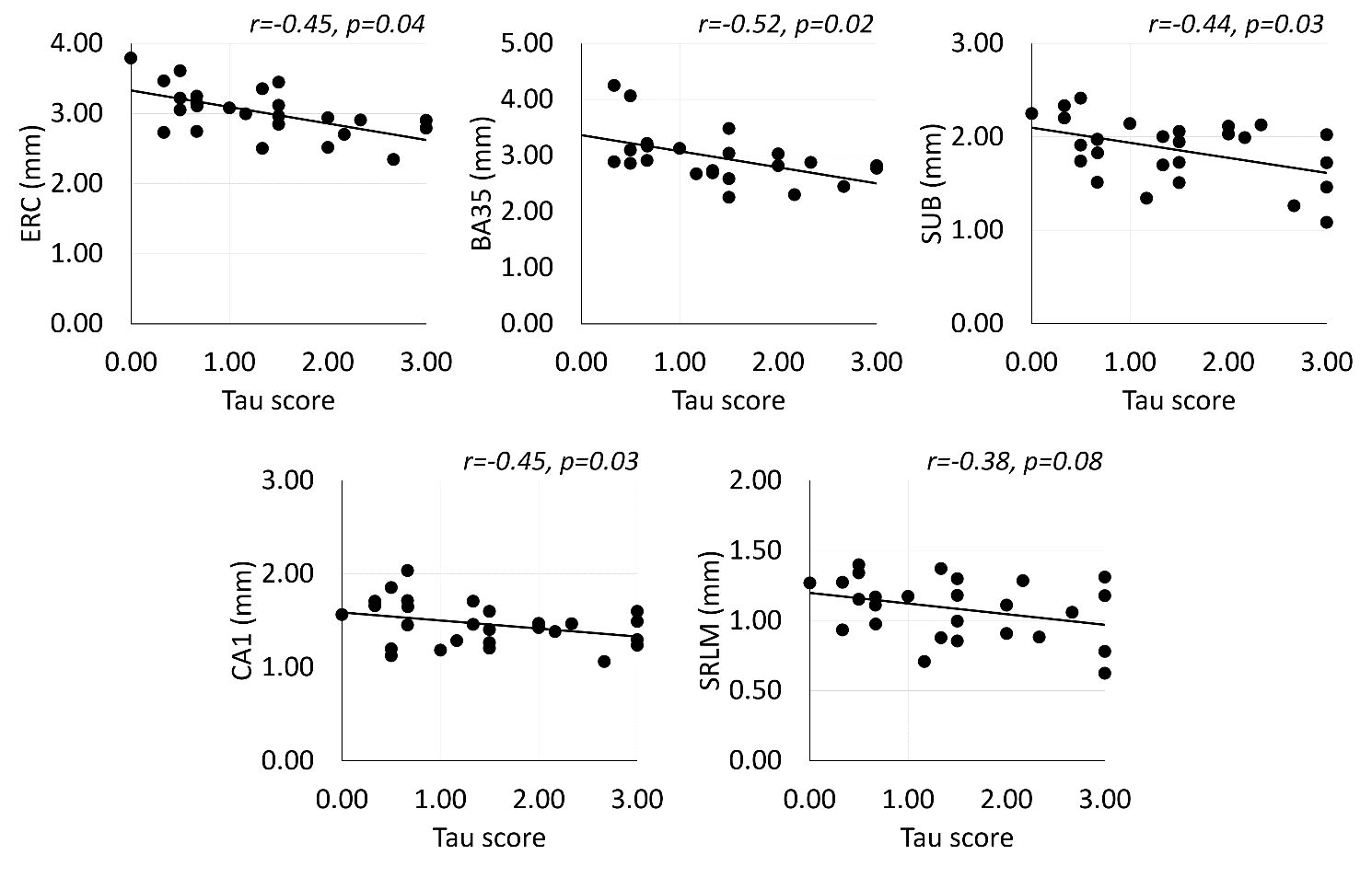


**References**

1. Adler DH, Wisse LE, Ittyerah R, Pluta JB, Ding S, Xie L, Wang J, Kadivar S, Robinson JL, Schuck T (2018) Characterizing the human hippocampus in aging and Alzheimer's disease using a computational atlas derived from ex vivo MRI and histology. Proceedings of the National Academy of Sciences 115: 4252-4257.

2. de Flores R, Wisse LE, Das SR, Xie L, McMillan CT, Trojanowski JQ, Robinson JL, Grossman M, Lee E, Irwin DJ (2020) Contribution of mixed pathology to medial temporal lobe atrophy in Alzheimer's disease. Alzheimer's & Dementia 16: 843-852.

3. Tustison NJ, Avants BB, Cook PA, Zheng Y, Egan A, Yushkevich PA, Gee JC (2010) N4ITK: improved N3 bias correction. IEEE Trans Med Imaging 29: 1310-1320. Doi:10.1109/TMI.2010.2046908 [doi].

4. Wisse L, Adler DH, Ittyerah R, Pluta JB, Robinson JL, Schuck T, Trojanowski JQ, Grossman M, Detre JA, Elliott MA (2017) Comparison of in vivo and ex vivo MRI of the human hippocampal formation in the same subjects. Cerebral Cortex 27: 5185-5196.
